# Structural and spectral characterisation of Er^3+^ and Nd^3+^ doped Ga-La-S-Se glasses

**DOI:** 10.1039/c8ra04795b

**Published:** 2018-08-02

**Authors:** A. Ravagli, N. G. Boetti, F. A. Guzman Cruz, G. A. Alzaidy, D. Pugliese, D. Milanese, D. W. Hewak

**Affiliations:** Optoelctronics Research Centre, University of Southampton University Road SO171BJ Southampton UK ar1e15@soton.ac.uk; Istituto Superiore Mario Boella Via P. C. Boggio 61 10134 Torino Italy; Umm Al Qura University, Department of Physics Makkah Saudi Arabia; Politecnico di Torino, INSTM Corso Duca degli Abruzzi 24 10129 Torino Italy; Consiglio Nazionale delle Ricerche, Istituto di Fotonica e Nanotecnologie Via alla Cascata 56/C 38123 Trento Italy

## Abstract

In this work, the spectroscopy of Er^3+^ and Nd^3+^ doped Se-GLS glasses was studied. A structural comparison between doped and non-doped samples was done to assess the differences between the glasses. For this comparison, Raman spectroscopy and thermal analysis were employed. The spectral properties of the samples were studied in order to identify the mechanisms responsible for quenching the fluorescence lifetime of the dopants. In particular, cross-relaxation and concentration quenching were observed in Nd^3+^ doped samples, whilst co-operative upconversion, radiation trapping and concentration quenching were observed in Er^3+^ doped samples. The results obtained demonstrated the fundamental role of the phonon energy in the mechanism of fluorescence. The low phonon energy of chalcogenides decreased the rate of non-radiative processes promoting co-operative upconversion. This effect could be exploited to design new lasers and sensitizers for solar energy harvesters.

## Abstract 2

Spectral characterisation of Er^3+^ and Nd^3+^ doped novel GLS-Se glass showing strong green fluorescence.

## Graphical abstract

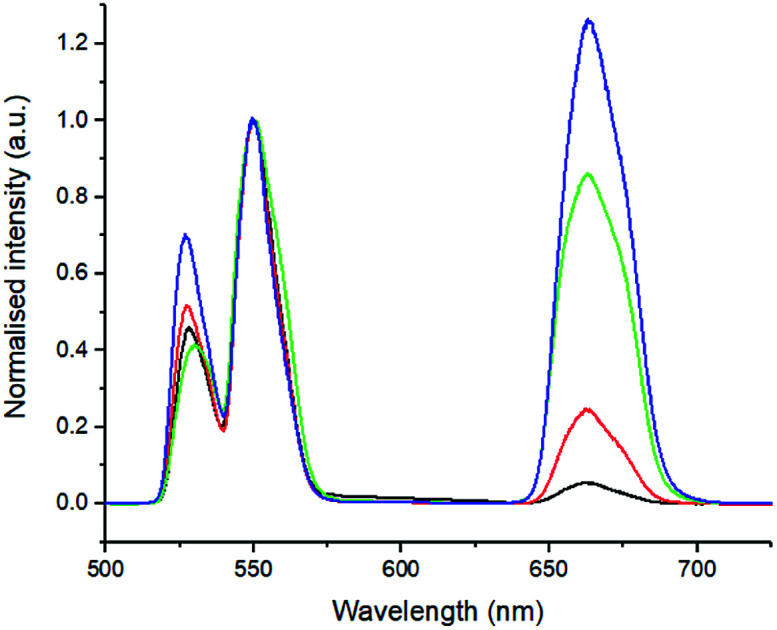

## Introduction

Chalcogenide glasses are amorphous materials widely used for infrared light transmission. Unlike conventional glasses, they do not contain oxides; they consist of the chalcogen elements S, Se and Te bonded with metals and semiconductors and forming covalent bonds.^1^ Their optical properties are strictly related to their chemistry. More in detail, the high atomic weight of the elements results in a low phonon energy, determining the large transparency in the infrared region.^2^ In addition, the large polarisability of the components results in a high refractive index between 2 and 3.^3^

Rare earth (RE) doping of chalcogenide glasses has been widely studied to develop knowledge on the employment of those glasses as sources for the emission of infrared light.^4^ In most amorphous materials, the electronic transitions related to infrared light emission occur over a small energy gap, which could be efficiently bridged by lattice vibrations in a non-radiative process. However, the characteristic energy of lattice vibration in chalcogenides is sufficiently low to allow infrared light emission.

The only chalcogenide glass fibre laser reported to date was demonstrated with Nd^3+^ doped GLS glass fibres operating at 1080 nm.^5^ Since then, considerable effort has been made to develop chalcogenide fibre lasers at longer wavelengths and using other chalcogenide glasses and dopants. However, the strong covalent character of chalcogenides acts on the spectroscopic properties by reducing the lifetime, increasing the pump power threshold and limiting the solubility of the rare earth ions.^6,7^

**Fig. 1:**
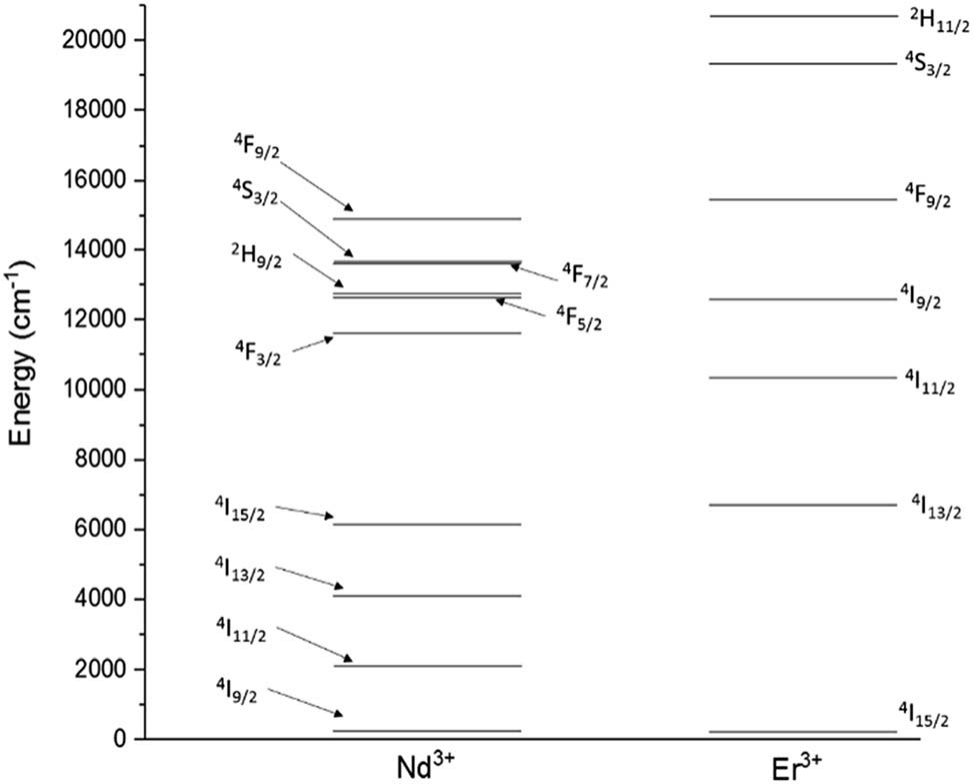
Energy level diagrams for Nd^3+^ and Er^3+^.

These issues could be overcome by manufacturing glasses in fibre form, where the fibre geometry can improve the pump efficiency, and by tailoring the chemistry of the glass. However, the performance of RE-doped materials, regardless of device geometry, is affected by processes which take place once the ions are pumped to the excited state.^7^ These include, as main cause of reduction in efficiency of the RE emission, detrimental cross-relaxation and co-operative upconversion (Fig. 1).

**Fig. 2:**
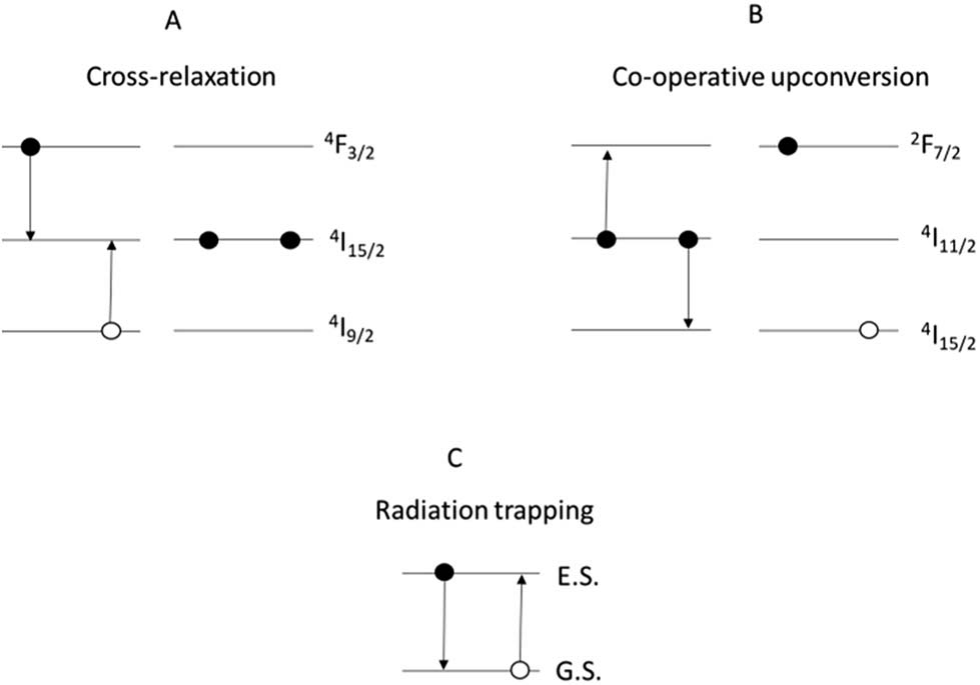
Mechanisms of: (A) cross-relaxation in Nd^3+^; (B) co-operative upconversion in Er^3+^; (C) radiation trapping. Note: for clarity, some of the energy levels have been omitted.

Cross-relaxation (Fig. 2A) is strong in materials containing Nd^3+^ ions and occurs when an ion in the energy state ^4^F_3/2_, instead of decaying radiatively, transfers part of its energy to a neighbouring ion at the ground state, resulting in both the ions being in the energy state ^4^I_15/2_.

Co-operative upconversion (Fig. 2B) is observed in Er^3+^ and occurs when two ions in the energy state ^4^I_11/2_ exchange energy, resulting in a doubly excited ion promoted to the level ^4^F_7/2_ and another at the ground state ^4^I_15/2_. The ions downconverted from the energy state ^4^I_11/2_ to the ground state by co-operative upconversion can reabsorb part of the emitted light released upon radiative decay. This effect is called radiation trapping (Fig. 2C).^8,9^ As a result of these effects, the discharge of the energy from excited states is delayed and the measured excited state lifetimes are lengthened. Lastly, an excessive concentration of dopant can result in the clustering of the dopant ions. This facilitates the energy transfer between ions decreasing the excited state lifetime and limiting the efficiency of light emission.

The aim of this work was to develop a better understanding of the dynamics governing energy transfer in the recently reported Se-GLS glass.^10^ In particular, cross-relaxation of Nd^3+^ ions and the combination of upconversion and radiation trapping of Er^3+^ ions were studied. The results were interpreted in order to evaluate Se-GLS glass as a potential rare earth host and a gain medium for fibre lasers and amplifiers. The lower phonon energy could facilitate the development of devices working in the mid and far infrared up to 15 μm. The large amount of upconversion and radiation trapping observed in Er-doped samples could be exploited for the improvement of the efficiency of photonic devices. In particular, radiation trapping could be tailored to decrease the pump power threshold of lasers.^11^ On the other hand, previous works on Er^3+^ doped light harvesting devices reported an improved efficiency due to strong upconversion.^12–14^ Er^3+^-doped chalcogenides as improved light harvesters could be considered for these studies in the future.

## Experimental

### Samples synthesis

The samples were synthesised following the procedure described in a previous work.^10^ Two series of glass hosts comprising high purity Ga_2_S_3_ (99.999%), Ga_2_Se_3_ (99.999%) and La_2_S_3_ (99.9%) were doped with Nd_2_S_3_ (99.9%) and Er_2_O_3_ (99.999%), respectively. The composition of the samples is reported in Table 1. The addition of Er^3+^ and Nd^3+^ was done substituting La^3+^ in order to maintain a constant level of rare-earth in the samples. The samples 40Se-GLS and 60Se-GLS were studied to investigate the effect of Se on the spectroscopic properties of the glass. The mixtures were prepared in a glovebox, homogenised and melted following a melt-quenching method. The powders were melted in amorphous carbon crucibles in order to avoid any reactivity between the melt and the vessel. The crucibles were placed in a quartz tube with a constant flow of dry-argon during the whole length of the process. The furnace employed for the melting process is a split tube-type, with the temperature ramp set at 10 °C min^−1^. The mixtures were baked for 3 h at 350 °C to eliminate volatile contaminants such as H_2_O, SO_2_ and SeO_2_. The temperature was then held at 1150 °C for 24 h to melt the mixtures and ensure a good homogenisation of the melt and the elimination of any dissolved gas. The furnace was located on top of tracks allowing the furnace to slide away from the melt during the quenching step. The quenching step occurred *via* heat exchange between the sealed system and the air, without any additional cooling aid. The samples were annealed at 490 °C with the temperature ramp set at 1 °C min^−1^ for both heating and cooling steps. During the annealing step, the glasses released any structural stress improving robustness and phase stability.

**Table 1:** Composition, characteristic temperatures, Weinberg parameter, density and dopant ionic density of the studied samples

Sample	Ga_2_S_3_ (wt%)	Ga_2_Se_3_ (wt%)	La_2_S_3_ (wt%)	Dopant (wt%)	*T*_g_ (±3 °C)	*T*_x_ (±3 °C)	*T*_m_ (±3 °C)	*K*_w_	Density (±0.001 g cm^−3^)	Dopant ionic density (10^19^ cm^−3^)
GLS^21^	70.00	—	30.00	—	553	660	815	0.131	—	—
20Se-GLS	38.60	24.66	36.74	—	522	682	789	0.202	—	—
01Nd_2_S_3_	38.31	24.77	36.78	0.14	527	687	791	0.186	4.249	1.91
05Nd_2_S_3_	38.57	25.00	35.82	0.60	528	684	793	0.192	4.228	8.06
1Nd_2_S_3_	38.40	25.02	35.30	1.26	530	692	795	0.210	4.245	16.10
2.5Nd_2_S_3_	38.71	24.49	33.70	3.11	527	694	790	0.211	4.233	21.70
5Nd_2_S_3_	38.64	24.53	30.46	6.36	528	689	792	0.202	4.269	85.20
01Er_2_O_3_	38.75	24.68	36.38	0.18	525	672	789	0.197	4.241	2.40
05Er_2_O_3_	38.44	24.81	36.11	0.64	526	678	790	0.204	4.263	8.61
1Er_2_O_3_	38.35	25.06	35.26	1.33	523	689	790	0.203	4.271	17.90
2Er_2_O_3_	38.50	24.75	34.26	2.49	531	638	782	0.137	4.207	35.10
40Se-GLS	20.21	45.15	33.63	—	500	663	763	0.214	—	—
1Er_2_O_3_-1	21.37	44.14	33.55	0.94	508	665	762	0.207	4.494	14.10
60Se-GLS	6.51	62.46	31.02	—	486	627	767	0.184	—	—
1Er_2_O_3_-2	6.37	6.19	30.71	1.03	485	635	775	0.194	4.679	15.00

### Samples characterisation

The phase of the samples was determined examining their Raman spectra. In principle, amorphous samples should be characterised by broad peaks within the same shift range of the crystalline form. The thermal characterisation was carried out with a Perkin-Elmer Diamond TG-DTA (Thermogravimetric-differential thermal analyser) to determine the characteristic temperatures, namely glass transition temperature (*T*_g_), crystallisation temperature (*T*_x_) and melting temperature (*T*_m_). The values measured were used to compare the samples with the relative non-doped glass. The density of the samples was assessed by the Archimedes' method using distilled water as immersion fluid. The spectroscopic characterisation included transmission spectra, emitted spectral lines and *P*^*n*^ curves. In *P*^*n*^ curves the pump power was plotted against the emitted intensity to determine the coefficient *n* representing the number of photons mediating the emission.^15^ The transmission spectra of polished samples were registered with a Cary 50 UV-visible spectrometer. A Renishaw “inVia” Raman spectrometer equipped with a laser with *λ* = 532 nm was used for Raman spectroscopy of Nd^3+^-doped samples. Due to the strong photoluminescence of Er^3+^ ions upon absorption of this laser line, only thermal analysis was used to characterise Er doped samples. For the measurement of the emitted spectral lines and the *P*^*n*^ curves, the pump lasers used for the experiments were operating at the wavelengths of 975 nm for Er^3+^ and 785 nm for Nd^3+^. The edge of each sample was irradiated by the pump light. The emitted light was collimated and focused into a spectrometer (Jobin Yvon iHR320) with the aid of appropriate optical elements. For the near-infrared region a Hamamatsu P4631-02 detector was used, whilst a Hamamatsu R928P detector was employed for the visible light emission. The signal was amplified with a lock-in amplifier (Stanford Research Systems SR830 DP lock-in amplifier). A similar set up was used for the determination of the excited state lifetime. For this measurement, the pumping light was modulated by a frequency generator and the signal was detected with an amplified detector (Thorlabs PDA10CS). The decay curves were recorded with a digital oscilloscope (Tektronix TDS350).

## Results and discussion

**Fig. 3:**
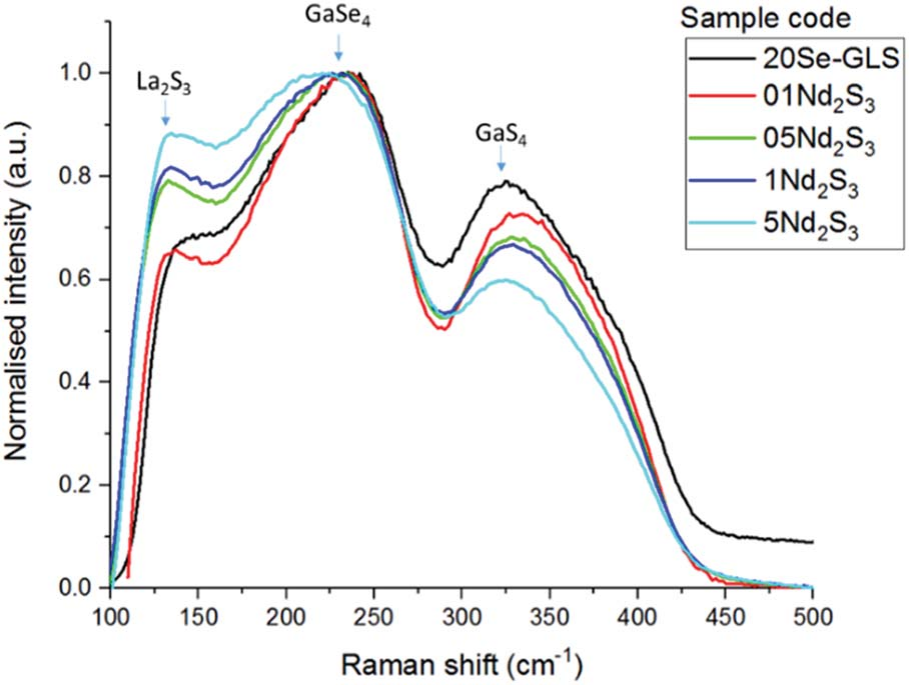
Raman spectra of undoped Se-GLS and Nd-doped samples.

Raman spectroscopy and thermo-gravimetric analysis were employed to study the structural variation of the glasses upon introduction of the dopants. Fig. 3 depicts the comparison between the normalised Raman spectra of Nd^3+^ samples and regular Se-GLS glass. The addition of incremental quantities of Nd_2_S_3_ resulted in the peak centred at 145 cm^−1^, assigned to La_2_S_3_ in previous studies, to increase in intensity as the concentration of Nd^3+^ increases.^16^ The enhanced broadness observed for the GaSe_4_ band between 200 and 300 cm^−1^ could be the result of the contribution of Nd_2_S_3_ to both the GaSe_4_ and La_2_S_3_ bands and the increased complexity of the structure due to the new component.^17,18^ The merging of the Raman bands of La_2_S_3_, Nd_2_S_3_ and GaSe_4_ also resulted in the decreased normalised intensity of the GaS_4_ band located between 300 cm^−1^ and 400 cm^−1^.

The characteristic temperatures of the un-doped and doped glass samples, measured to further investigate their structure, are summarised in Table 1. The substitution of La^3+^ with Nd^3+^ and Er^3+^ did not change dramatically the characteristic temperatures. The increased *T*_g_ and *T*_m_ observed for all the samples could be due to the higher melting point of the dopants and their different chemistry with respect to La_2_S_3_.^19,20^ On the other hand, the melting point did not vary substantially, confirming that the doped samples did not undergo changes in structure. The Weinberg's factor *K*_w_ was calculated as:1
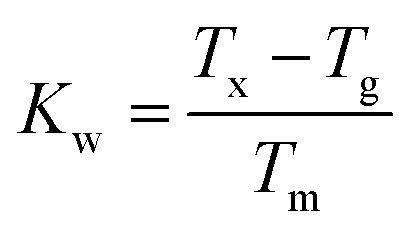
to assess the thermal stability of the samples.^21^ The values of *K*_w_ assessed for the doped samples were found to be slightly smaller than those reported for the non-doped glasses as a result of the contraction of the *T*_x_ − *T*_g_ gap. However, it remained well above the *K*_w_ of the Se-free GLS glass used to build the only reported chalcogenide fibre laser.^5,22^ Therefore, with these improved thermal properties, the Se modified GLS could also be suitable for fibre drawing with the goal of demonstrating an improved rare earth doped chalcogenide fibre laser.

### Spectroscopy of neodymium-doped samples

**Fig. 4:**
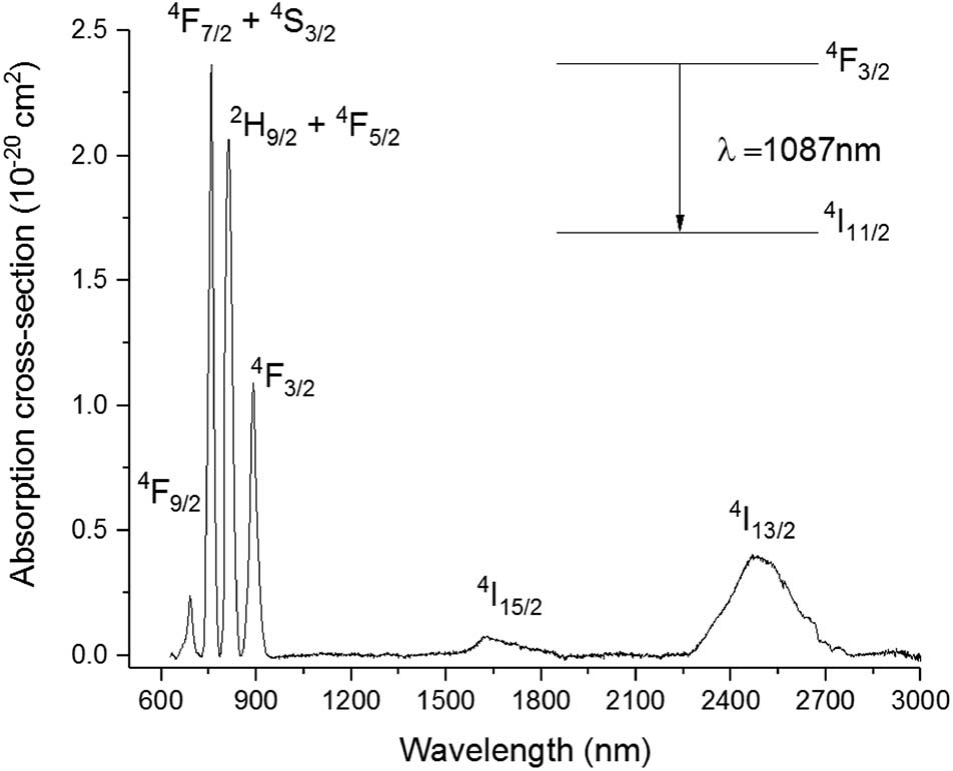
Absorption cross-section spectrum of 01Nd_2_S_3_ glass. Note: absorption by level ^4^I_11/2_ occurring at 5.30 μm was not included.

Fig. 4 shows the absorption cross-section spectrum of 01Nd_2_S_3_ glass. The absorption cross-section values were calculated from the transmission spectrum using the formula:2
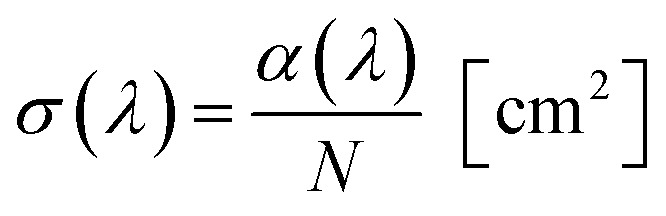
where *α*(*λ*) is the absorption coefficient in cm^−1^ and *N* is the atomic concentration of dopant in cm^−3^. The main Nd^3+^ levels, corresponding to the ground state absorption of Nd^3+^, are also shown in Fig. 4.

**Fig. 5:**
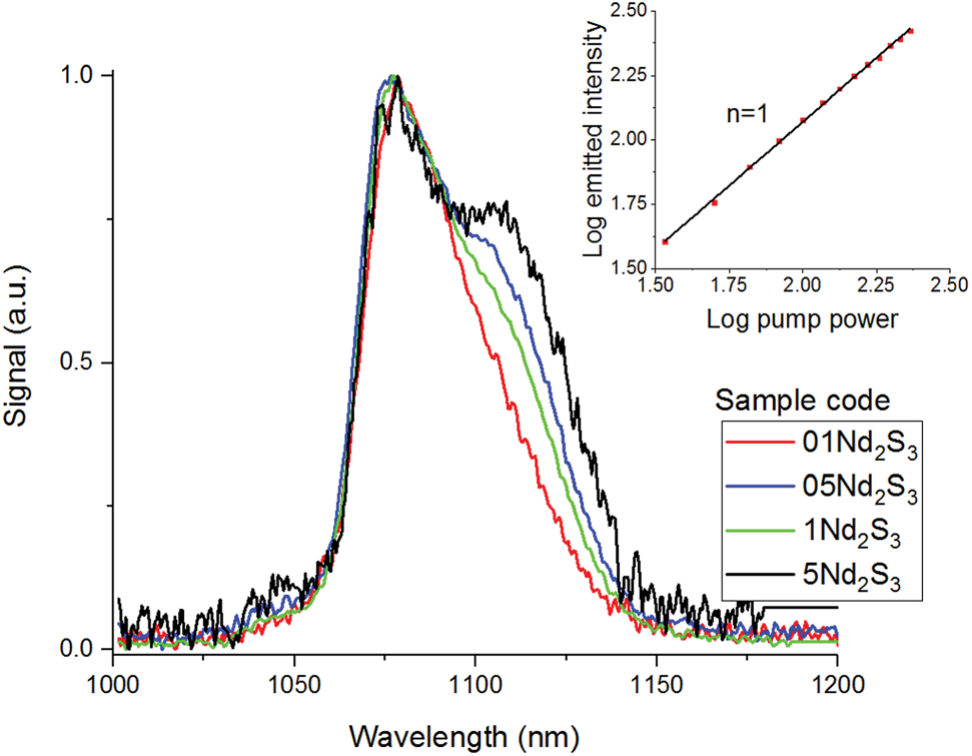
Emission spectra of samples 01Nd_2_S_3_, 05Nd_2_S_3_, 1Nd_2_S_3_ and 5Nd_2_S_3_ and *P*^*n*^ curve for the transition ^4^F_3/2_ → ^4^I_11/2_ of the sample 05Nd_2_S_3_. For clarity, the spectrum of 2.5Nd_2_S_3_ has been omitted.

The *P*^*n*^ curve for the transition ^4^F_3/2_ → ^4^I_11/2_ was determined to study the dynamics of the emission process by increasing the pump power from 34 to 231 mW. In principle, the intensity of the emitted light should be proportional to the incident pump power to the power of *n*. The exponent *n* represents the number of photons mediating the emission. In absence of any phenomenon but radiative relaxation contributing to the emission, the exponent should be equal to 1. The value of *n* can be determined from the so-called log–log plot, in which the logarithm of the pump power is plotted against the logarithm of the intensity of the emission. The slope of the resulting straight line represents *n*.^23^ As shown in the inset of Fig. 5, the slope of the log–log plot for the transition ^4^F_3/2_ → ^4^I_11/2_ was 1, excluding any interference by other phenomena with the emission. The peak intensity of the emission band was found at 1087 nm, whilst it was 1080 nm for the Se-free GLS glass.^5^ This shift is known as the “nephelauxetic effect” and it was observed in other glasses upon changes in composition.^4^ In particular, the addition of ionic compounds shifted this emission band towards shorter wavelengths, whilst the addition of covalent compounds had the opposite effect, shifting the emission band towards longer wavelengths.^24^ Since Ga_2_Se_3_ has a covalent character more pronounced than Ga_2_S_3_ and La_2_S_3_, the red-shift of the ^4^F_3/2_ → ^4^I_11/2_ in Se-GLS can be attributed to this effect. Furthermore, the broadening of the emission band has been observed as the concentration of Nd^3+^ was increased (Fig. 5). In particular, the broadening occurred preferentially towards longer wavelengths. The cause of this observation could be the chemistry of the site occupied by the rare earth, which has been recognised as a source of inhomogeneous broadening.^7^

**Fig. 6:**
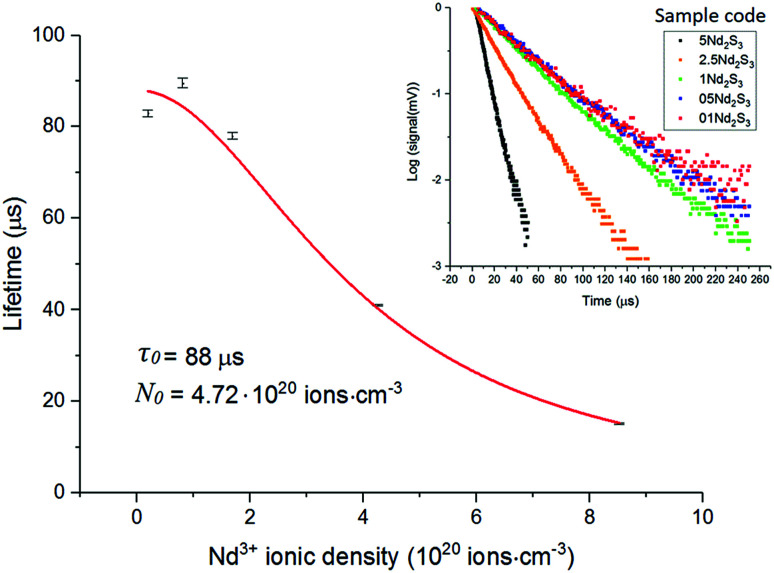
Excited state Nd^3+^:^4^F_3/2_ lifetime for the samples 01Nd_2_S_3_, 05Nd_2_S_3_, 1Nd_2_S_3_, 2.5Nd_2_S_3_ and 5Nd_2_S_3_. The filled squares represent the experimental data, while the continuous line is the fitting to eqn (3). The inset shows the decay curves on a log scale of the ^4^F_3/2_ level of Nd^3+^-doped samples.

In light of the nephelauxetic effect, the preferential broadening towards longer wavelengths may suggest that Nd^3+^ occupies increasingly covalent sites as the doping level grows, justifying the larger FWHM (Full Width at Half Maximum) in comparison with other oxide glasses.^24^ However, further investigation such as XPS (X-ray Photoelectron Spectroscopy) may give information to confirm these hypotheses. The effect of the concentration of the dopant on the excited state lifetime is shown in Fig. 6. The shortening of the measured excited state lifetime for increasing concentrations of Nd^3+^ was attributed to the occurrence of cross-relaxation. As depicted in Fig. 2A, cross-relaxation in Nd^3+^ takes place when an ion at the excited state transfers energy to an ion at the ground state.^7^ As this quenching mechanism is phonon-assisted, the final result consists of the pump light being converted into heat. Thus, the phonon energy of the material plays a key role on the cross-relaxation rate.^25^ Materials with a high phonon energy (*i.e.* oxide glasses) are characterised by a high cross-relaxation rate which leads to a fast increase in temperature of the pumped material. The high ionic density and short distances between the dopant ions in the host accentuate the occurrence of cross-relaxation. The quenching effect of the dopant concentration can be quantified from the equation:^9^3
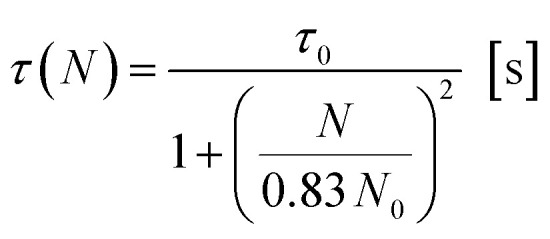
where *τ*(*N*) is the measured lifetime, *τ*_0_ is the theoretical lifetime in absence of any cross-relaxation (*i.e.* single ion in the host material), *N* is the ionic concentration in cm^−3^ and *N*_0_ is the quenching concentration. The parameters *τ*_0_ and *N*_0_ were found fitting the experimental data with the least square method. The fitting resulted in a theoretical lifetime of 88 μs and a quenching concentration of 4.7 × 10^20^ ions per cm^3^.

The value of lifetime agreed with previous studies on Nd^3+^ doped chalcogenide glasses, but was significantly shorter than in oxide glasses.^5,24,26,27^ This reduction of the lifetime is characteristic of optical materials with high refractive index and consequently high *σ*_em_*τ* product.^7^ On the other hand, the critical concentration found for Se-GLS glass resulted to be comparable to those reported for silicates, phosphates and other well characterised oxide and halide glasses.^7^ The large solubility of lanthanides in GLS-based glasses arise from the interaction between the empty p-orbital of Ga^3+^ and the S^2−^, leading to the formation of a dative bond.^28^ This approach has been applied to increase the solubility of rare earths in silica glass by adding Al^3+^.^29^ Additional stabilisation may derive from the large content of La^3+^ in GLS matrices, which can be easily substituted with other rare earth ions. A similar interaction is conceivable in Se-GLS due to the similar chemistry of Se and S. However, the balance between the ionic character of La_2_S_3_ and the covalent Ga_2_S_3_ is key to the glass formation and an excess of Se leads to devitrification. As a result, attempts to synthesise GLSe (*i.e.* full substitution of S by Se) were found unsuccessful and glass formation of Se-GLS occurs in a rather narrow range of concentrations of La^3+^.^10,30^

### Spectroscopy of erbium doped samples

The absorption cross-section spectrum of Er^3+^ obtained from the absorption spectrum of the sample 05Er_2_O_3_ is shown in Fig. 8. This is compared to the emission cross section calculated as reported in past works on Er^3+^-doped GLS glass.^31^ To focus on the study of the transition Er^3+^:^4^I_13/2_ → ^4^I_15/2_, which emits a spectral band, centred at 1537 nm, a slightly different approach was adopted. In particular, in erbium-doped materials pumped at 980 nm upconversion from the level ^4^I_11/2_ to ^2^H_11/2_ is commonly observed. This mechanism is found to be stronger when the non-radiative decay ^4^I_11/2_ → ^4^I_13/2_ is slow.

**Fig. 7:**
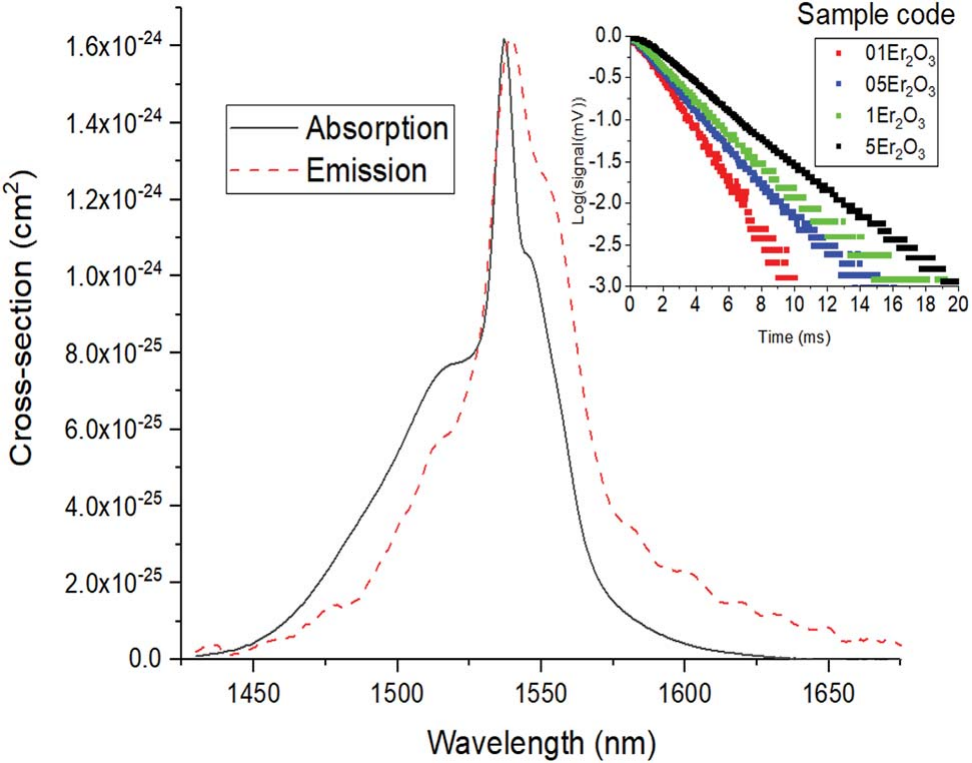
Absorption and emission cross section of the transition ^4^I_13/2_ → ^4^I_15/2_ of Er^3+^. The inset reports the measured decay curves for the transition ^4^I_13/2_ → ^4^I_15/2_ of Er^3+^-doped samples in logarithmic scale.

The phonon energy affects the speed of this decay. The latter is low for chalcogenide glasses, thus determining a large upconversion.^4,32^ As depicted in Fig. 2B, this process leads to a doubly-pumped ion and an ion at the ground state. The ion decayed to the ground state is thus capable of reabsorbing a portion of the emitted light with energy matching the ground state absorption (Fig. 2C). The consequences of this effect are an exceedingly long lifetime (Fig. 7) and broadened emission bands (Table 2).^33,34^ In principle, the issue can be overcome by milling the glass and recording the emission spectra and lifetimes of the powder.^35^ However, the large surface area of the powder makes the material prone to absorption of moisture from the air and oxidation. This would result in a change in the composition of the glass over the time and the measurement of the spectral properties of a glass with a large content of oxygen and OH^−^. The effect of reabsorption on the measured lifetimes can be quantified as:^8^

4 *τ*_R_ = *τ*_0_(1 + *Nσ*_abs_*l*)[s]

where *τ*_R_ is the lifetime in conditions of reabsorption and *l* is the average absorption length. From this equation it can be highlighted that the observed lifetimes tend to increase as the concentration of the dopant grows. However, the increase in concentration of the dopant should result in shorter ion–ion distances and thus more energy transfer occurs with a faster depopulation of the excited states.

**Table 2:** Summary of the spectral characterisation of Er-doped samples.^a^

Sample	^4^I_13/2_*τ*_m_ (ms)	^4^I_13/2_*τ*_JO_ (ms)	*Ω*_2_ (10^−21^ cm^2^)	*Ω*_4_ (10^−21^ cm^2^)	*Ω*_6_ (10^−21^ cm^2^)	^4^I_13/2_ FWHM (nm)	^4^F_9/2_ FWHM (nm)	^4^S_3/2_ FWHM (nm)	^2^H_11/2_ FWHM (nm)
01Er_2_O_3_	3.61	—	—	—	—	50.0	22.2	14.4	16.0
05Er_2_O_3_	4.38	—	—	—	—	49.6	23.2	14.2	15.6
1Er_2_O_3_	5.07	3.37	2.218	9.309	9.305	56.1	28.0	16.4	18.8
2Er_2_O_3_	5.74	—	—	—	—	41.9	26.0	13.8	15.0
1Er_2_O_3_-1	5.96	3.26	1.799	6.815	8.160	55.8	26.2	15.6	16.0
1Er_2_O_3_-2	6.87	2.74	0.398	4.201	9.205	41.5	22.6	n/d	n/d

a n/d peaks are due to band gap absorption

Both these contributions can be taken into account by multiplying eqn (3) and (4) giving:^8^5
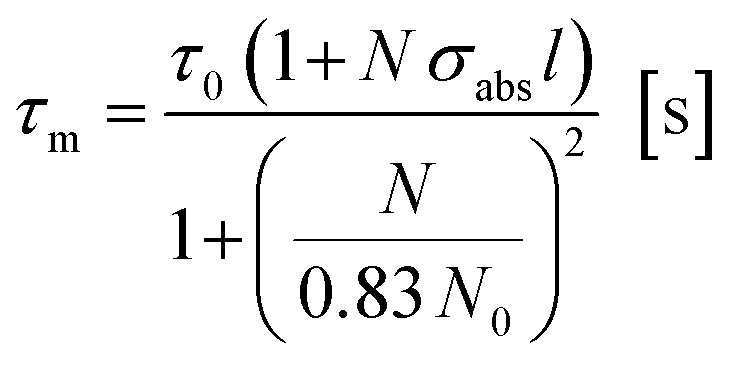


**Fig. 8:**
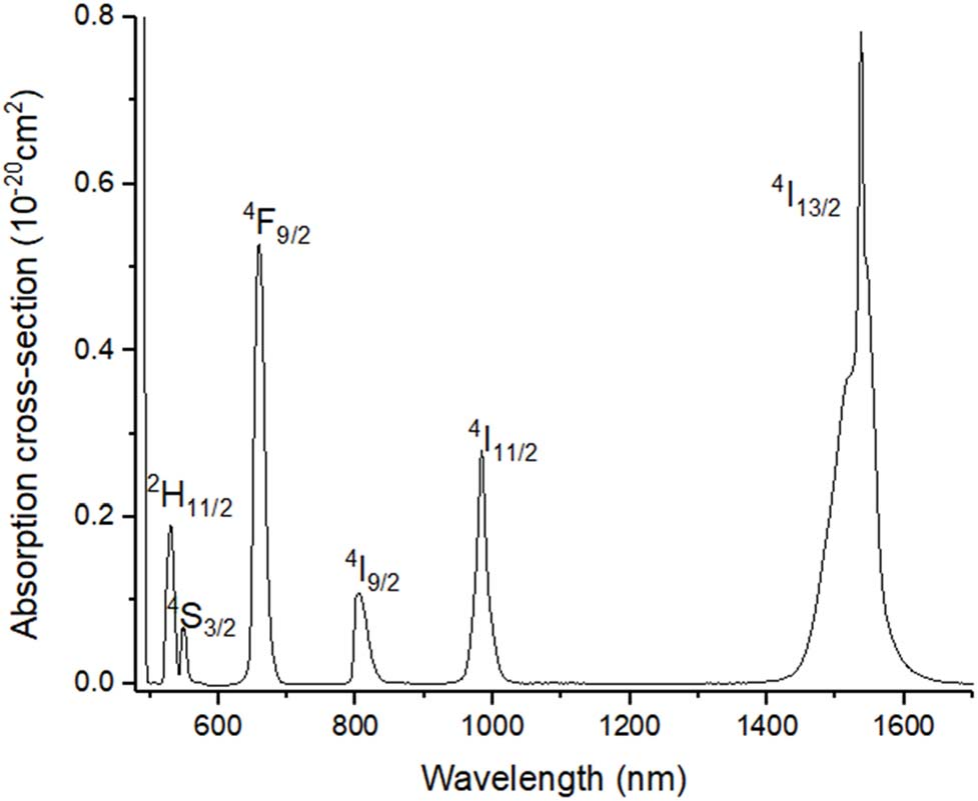
Absorption cross-section spectrum of Er^3+^-doped glass (sample 05Er_2_O_3_).

**Fig. 9:**
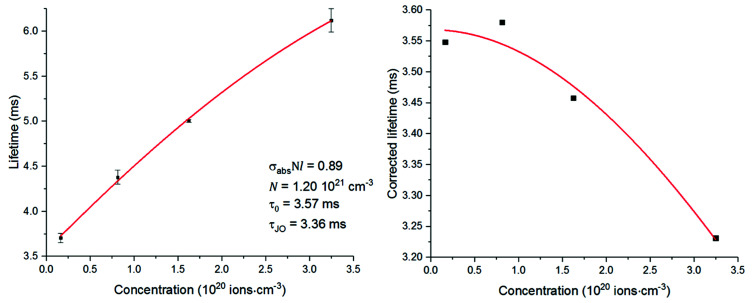
Left, measured excited state lifetimes for the transition ^4^I_13/2_ → ^4^I_15/2_ fitted to eqn (5); right, excited state lifetimes after correction fitted to eqn (3).

The lifetimes measured for different concentrations can be fitted to this equation determining *σ*_abs_*l*, *τ*_0_ and *N*_0_ as fitting parameters. It must be noted that the equation can be applied only if *Nσ*_abs_*l* < 1. Once the fitting parameters have been determined, it is possible to eliminate the effect of reabsorption from the experimental data. The true lifetimes can be fitted to eqn (3) keeping the same *τ*_0_ and *N*_0_ found with eqn (5). The plot on the left of Fig. 8 shows the trend of the lifetimes of the transition ^4^I_13/2_ → ^4^I_15/2_ measured for bulk samples and the fitting to eqn (5). The growing trend suggested a large extent of reabsorption masking the quenching effect of the concentration. The elimination from the measured lifetimes of the reabsorption effect gave the expected decreasing trend typical of concentration quenching (right-hand side of Fig. 8). The equations were fitted to the experimental data with a good accuracy, as demonstrated by *R*^2^ = 0.997. *τ*_0_ proved to be in good agreement with the radiative lifetime calculated with the Judd–Ofelt analysis (*τ*_JO_), as described in previous works.^36–38^ Further investigations on the visible spectra were carried out observing that the relative intensity of the emission at 665 nm became progressively prominent as the concentration of Er^3+^ ions grew (Fig. 9). As shown in Fig. 8, the energy levels ^2^H_11/2_ and ^4^S_3/2_ emitting light at 530 and 550 nm are close to the electronic edge. This suggests that part of the radiation with wavelengths 530 and 550 nm could have absorbed by the electronic edge since the electronic configuration of the dopant ions fell into the band gap absorption reducing the intensity of the emission. Additional considerations have been done to assess the contribution to the intensity of the emission at 1537 nm from upconverted ions decaying to the ^4^I_13/2_ level. For this analysis the *P*^*n*^ curves of the emissions at 530, 552, 663 and 1537 nm have been measured to determine the value of *n* for each emission (Fig. 9). As expected, for all the visible emissions the value of *n* was found around 2, proving that two pump photons were necessary to mediate the emission (*i.e.* upconversion). On the other hand, the value of *n* for the emission at 1537 nm was found close to 1, which suggested a negligible contribution to this emission by higher-lying energy levels. Indeed, the contribution of the decay from higher-lying levels to this emission would give a value of *n* closer to 2 since two pump photons would be needed for the phenomena to take place.

**Fig. 10:**
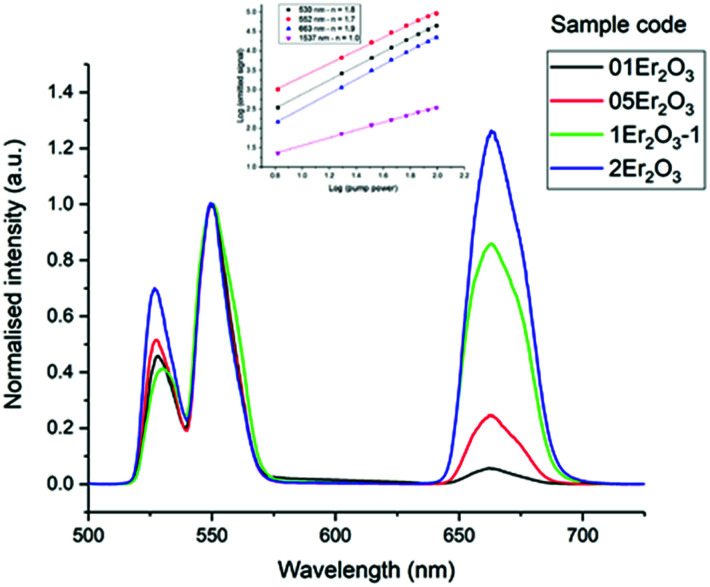
Er^3+^ visible emission spectra of the Er-doped Se-GLS glasses. The inset shows the *P*^*n*^ curves for sample 05Er_2_O_3_. Note: the intensities were normalised by the peak at 553 nm.

As an insight into future work, the emission corresponding to the transition ^4^I_13/2_ → ^4^I_15/2_ was studied in samples with higher amount of Ga_2_Se_3_ but comparable doping level of Er_2_O_3_, around 1 mol% (Table 2 and Fig. 10).

**Fig. 11:**
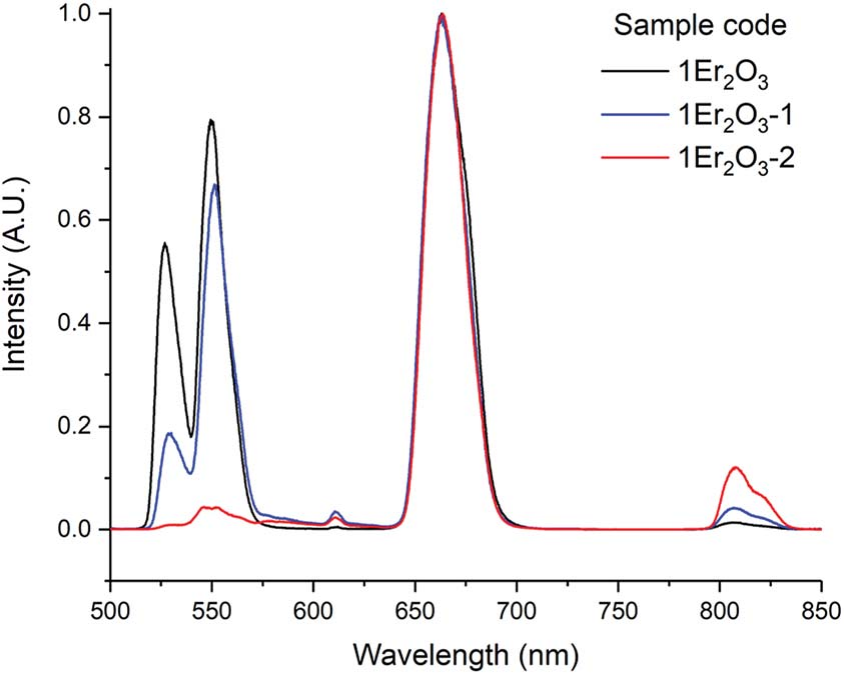
Emission spectra of the transition ^4^I_13/2_ → ^4^I_15/2_ for the Er^3+^ ion doped samples.

The measured lifetimes were observed to increase as the concentration of Se in the glass grew, confirming the relationship between phonon energy and reabsorption. Indeed, for higher content of Se the phonon energy of the material and the rate of non-radiative decay decreased, thus more upconversion occurred. This hypothesis is also supported by the decrease of *τ*_JO_ due to the increase of the refractive index. The growing gap between *τ*_JO_ and *τ*_m_ could also prove the correlation between the phonon energy and the reabsorption, as described above (Fig. 11).

### Comparison with other materials

A comparison between the spectral properties of the Se-GLS and other glasses is summarised in Table 3. For both Er and Nd doped glasses similar observations can be made. In particular, chalcogenide glasses exhibited shorter lifetimes than oxide-based materials due to their higher *σ*_em_*τ* product.^7^ In addition, the emission peaks were shifted towards longer wavelengths due to the nephelauxetic effect. The lifetimes for doped Se-GLS were found to be longer than those for doped GLS glass. This observation could be due to the improvements made over the last few year on the purity of the precursors and the fabrication of Se-GLS. Indeed, a lower amount of impurities can improve significantly the excited state lifetime.^39^ In the case of Er^3+^, it should considered that the Se-GLS samples were fabricated with Er_2_O_3_. For Er doped GLS and Ge-Ga-As-S, Er_2_S_3_ was used which contributed to increase the refractive index and decrease excited state lifetime. On the other hand, Nd doped Ga-Na-S and Ga-Ge-As-S exhibited longer lifetimes and the emission peak at shorter wavelengths than Nd doped Se-GLS. Due to the absence of Se, these materials have a less covalent character which could explain these differences. However, Ga-Na-S are strongly hygroscopic and the large amount of water absorbed is detrimental to the spectral properties. On the other hand, Ge-Ga-As-S glasses inferior mechanical properties than Se-GLS as reported in past works.^40^

**Table 3:** Comparison between Se-GLS and other rare earth hosts

Glass	Nd^3+^ doping		Er^3+^ doping	
	^4^F_3/2_ → ^4^I_11/2_		^4^I_13/2_ → ^4^I_15/2_	
	*τ* (μs)	Emission peak (nm)	*τ* (ms)	Emission peak (nm)
Se-GLS	88	1087	3.2	1537
GLS^5,41^	61	1078	2.3	1537
Ga-Na-S^5^	109	1080	—	—
Ga-Ge-As-S^5,42^	90	1077	2.33	1540
Silicate^7^	500	1060	10.0	1535
Phospate^7^	334	1055	7.1	1534
ZBLAN^7^	394	1049	9.4	1530

## Conclusions

A thorough investigation on the spectral properties of Er^3+^ and Nd^3+^ doped Se-GLS glasses was carried out. The doped samples did not undergo substantial structural changes in comparison to the non-doped materials. Indeed, the Raman shifts of Nd doped samples were in good agreement with these reported for standard Se-GLS glasses. In general, the thermal stability decreased as a consequence of unchanged *T*_m_ and slightly higher *T*_g_. The largest change in *T*_g_ was found as high as 10 °C for the sample 2Er_2_O_3_. This observation does not indicate significant changes in the structure of the glass even at high levels of doping.

The spectral properties of the two series of samples were investigated to determine the dynamics involved in the emission of light and further evaluate Se-GLS as a rare earth host. As expected, the transition ^4^F_3/2_ → ^4^I_11/2_ of Nd doped samples was affected by cross-relaxation, becoming more effective as the concentration of the dopant increased. Due to the strong covalent character of the glass, the emission peak was found at 1087 nm against 1060 nm usually found for silica.^24^ A theoretical lifetime *τ*_0_ as high as 88 μs was estimated in agreement with other reported chalcogenide glasses.

The study of the spectral properties of Er doped samples required a different approach due to the occurrence of radiation reabsorption. Indeed, the ^4^I_13/2_ → ^4^I_15/2_ transition was found to show an unusually long measured lifetime due to a large extent of emitted light reabsorption.

The impact of reabsorption was assessed fitting the measured lifetimes with eqn (5), which resulted in a *τ*_0_ similar to the one predicted with Judd–Ofelt analysis. A mechanism of reabsorption was formulated accounting for the upconversion and the growing lifetime for high concentration of the dopant. In particular, the transition ^4^I_11/2_ → ^4^I_13/2_ of Er^3+^ is a non-radiative process mediated by phonons. Due to the low phonon energy of the glass, this decay was rather slow and promoted the upconversion with the transition ^4^I_11/2_ → ^2^H_11/2_.

This work assessed the suitability of Se-GLS glass as an optically active material for applications such as lasers, signal amplifiers and sensing, as demonstrated for Nd:GLS glass in the past.^5^ The large reabsorption observed in Er doped materials could be used to improve the performance of existing devices. For instance, it was proposed that reabsorption could be tailored in order to act as an energy-storing process decreasing the pump power threshold of lasing crystals and glasses.^11^ In addition, the large amount of upconversion achievable with Er^3+^ was studies in previous works to improve the efficiency of light harvesters.^12–14^

## Conflicts of interest

There are no conflicts of interest to declare.

## Supplementary Material
